# Strength in numbers: how multimerization drives HpHb uptake by CD163 scavenging receptor

**DOI:** 10.1038/s41467-025-67812-3

**Published:** 2025-12-24

**Authors:** Omar De Bei, Barbara Campanini

**Affiliations:** 1 Department of Medicine and Surgery, University of Parma, Parma, Italy; 2 Department of Food and Drug, University of Parma, Parma, Italy

**Keywords:** Biochemistry, Structural biology, Cryoelectron microscopy

## Abstract

CD163 is a macrophage scavenger receptor central to the clearance of haptoglobin-hemoglobin (HpHb) complexes and the resolution of inflammation. In a remarkable example of near-simultaneous scientific progress, three complementary studies published in Nature Communications^1–3^ have resolved the near-atomic resolution structure of the full extracellular domain of human CD163 complexed with HpHb, primarily utilizing cryo-electron microscopy (cryo-EM). The most significant and unified conclusion emerging from this trio of papers is that CD163’s scavenging activity is fundamentally dependent on its ability to form functional multimers.

Scavenger receptors (SRs) represent a heterogeneous superfamily whose members share the common function of identifying and removing unwanted entities, be pro-oxidants (like LDL and haptoglobin-hemoglobin, HpHb, complexes), damaged proteins or microbial cells^4^. The Cluster of Differentiation 163 (CD163) receptor is a member of such superfamily, characterized by a small intracellular domain, a transmembrane portion that spans the membrane once, and a complex ectodomain composed by nine type B SR cysteine-rich (SRCR) domains^5,6^. In the juxta membrane region, a Ser-Arg-Arg-Ser sequence allows proteolysis by ADAM 17 with release of the soluble form of the receptor (sCD163)^7^. CD163 expression is typically observed in the monocyte/macrophage lineage where it is predominatly found in the M2 subpopulation, thus becoming a specific marker for these cells^4^. CD163 expression is induced by anti-inflammatory stimuli (e.g., IL-10) and by glucocorticoids while it decreases in the presence of pro-inflammatory stimuli^8^. CD163 is broadly involved in immune surveillance and hemoglobin (Hb) degradation, the latter being probably the better characterized function of the receptor. Indeed, in 2001 Moestrup and collaborators identified CD163 as the “Hb SR” that can recognize, internalize, and degrade haptoglobin (Hp)-bound Hb, thus contributing to its removal from circulation and to the management of heme-induced oxidative stress^9^. While over time other functions for CD163 have emerged, like its involvement in tumor development mediated by scavenging of TWEAK^10^ or its contribution to innate immunity through bacteria binding and immune system activation^11^, its role in Hb scavenging has remained the most thoroughly studied. The calcium dependence of ligand binding was evident from the very beginning, concomitant with the discovery of the receptor’s role in HpHb scavenging^9^. Shortly thereafter, the specific contribution of SRCR domains to ligand recognition and binding was elucidated^12^. Nevertheless, the subtle molecular details of the receptor structure, ligand recognition, and internalization have remained thus far elusive.

Remarkably, 2025 witnessed the publication of four journal articles^1–3,13^ reporting the cryo-electron microscopy (cryo-EM) structures of the full-length receptor, three of which appeared in *Nature Communications*. Despite the slightly different approaches used by the four groups to deal with fundamental unanswered questions about this receptor (which is its oligomeric state? Does binding to different Hp haplotypes differ? How do calcium ions mediate ligand binding?) they overall converge to a very coherent picture that not only clarifies the mechanism of HpHb scavenging but also convey some general features of CD163 function that helps in understanding its promiscuous functions.

All the studies have exploited cryo-EM whose inherent ability to detect distinct protein conformations has enabled the structural characterization of such large and inherently flexible macromolecular complexes that are often inaccessible to crystallography or NMR. A consistent observation across all reports is the receptor’s ability to assemble into dimers and trimers, with the trimeric form emerging as the potential physiologically most relevant state. Oligomer formation involves the assembly of a stable, globular core composed of the SRCR5–9 domains from the individual protomers, while the SRCR1–4 domains extend outward as rod-like segments. The interaction with the HpHb ligand occurs precisely through these protruding regions emanating from the central core (Fig. 1a).

**Fig. 1:**
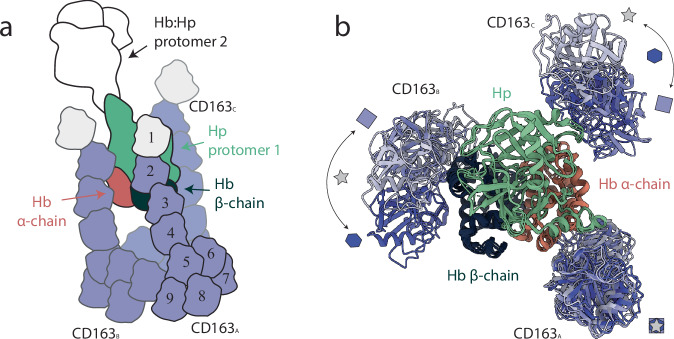
Comparison of CD163–HpHb structures obtained using cryo-EM. **a** Schematic overview of the regions of the CD163-HpHb complex resolved across the three cryo-EM studies. Colored segments indicate the portions of the complex that were structurally determined in each work, while white areas represent unresolved or missing density. The nine SRCR domains of CD163 are shown as numbered modules (1–9). Domains of CD163 protomers A (CD163_A_), B (CD163_B_), and C (CD163_C_) are shown in violet shades. Hp, and the Hb α- and β-chains are colored green, red, and dark blue, respectively. **b** Structural comparison of the SRCR1–4 regions of CD163 in the three cryo-EM studies. The color code is as in panel A. The HbHp complex and the SRCR1–4 regions of CD163_A_ were used as reference for structural alignment across the three structures. Receptors are colored according to the source publication: violet marked with a square^2^, lighter violet marked with a star^3^, and darker violet marked with a hexagon^1^. The overlaid models highlight conformational variability and provide a direct comparison of receptor conformations. The following PDB codes were used to generate the figure: 8XMP^3^, 9FNO^2^ and 9HEK^1^.

Because cryo-EM reconstructions rely on the alignment of identical particle views, conformational flexibility often leads to incomplete density maps, where highly mobile regions remain unresolved. This phenomenon is evident in all reported structures of the CD163–HpHb complex, which consistently lack one half of the HpHb dimer as well as the N-terminal SRCR1 domain of all receptor subunits (shown in white in Fig. 1a). In addition, other SRCR domains are partially or completely missing in different reconstructions, further reflecting the intrinsic flexibility of the receptor ectodomain.

Beyond the loss of density due to conformational variability, a comparative analysis of the deposited models reveals a common structural core shared across all reconstructions. This stable region comprises a single HpHb protomer— formed by one β-chain of Hp bound to a Hb dimer − together with the SRCR2–4 domains of one CD163 protomer, conventionally designated as protomer A. When the structures are aligned using this core, and only the SRCR2–4 segments of the receptor trimer are displayed, it becomes apparent that the remaining two protomers (B and C) adopt distinct relative orientations toward the HpHb complex (Fig. 1b). This observation suggests that, although the trimeric core formed by SRCR5–9 is generally considered stable, it nevertheless allows a degree of mobility among the protomers, which may persist even upon ligand engagement and potentially contribute to a dynamic interaction with other ligands.

The discovery of the trimer prompted the authors to investigate how this oligomeric state forms and how its formation affects the protein’s scavenging activity. Interestingly, all groups identified the same narrow region of the receptor as crucial for oligomerization. In particular, they converge on the idea that the SRCR7 domain of one protomer interacts with the SRCR9 domain of a second protomer, forming the key interface that stabilizes the oligomer. In this region, the interaction is mediated by two mirrored determinants, each consisting of a triad of carboxylate-containing residues (forming a calcium-binding site) and a lysine from the opposite protomer. Specifically, Lys811 (SRCR7) interacts with D955, D956, and E1022 (SRCR9), while Lys1021 (SRCR9) interacts with D745, D746, and E812 (SRCR7).

In this context, a key finding consistently reported across all studies was the identification of calcium as a crucial factor in stabilizing the oligomeric state. Although the residues mentioned above had already been proposed as putative calcium-binding sites^14^, their role in oligomerization had never been established. Notably, the different resolutions of the maps allowed the positioning of calcium ions with different degree of confidence, allowing in all cases to unveil their functional role.

The promising identification of the key determinant for receptor oligomerization prompted three out of four groups to engineer constructs aimed at disrupting oligomer formation, each employing approaches of varying sophistication^1–3^. In contrast, only Edzerodt and colleagues exploited a coiled-coil motif to stabilize the receptor trimer. Collectively, these molecular tools have been instrumental in dissecting the impact of oligomerization on ligand binding and internalization.

Consistently across studies, two main conclusions emerged. First, calcium is essential even for the basic receptor–ligand interaction: monomeric mutants displayed no binding to HpHb in the absence of calcium or in the presence of other divalent cations. Second, trimer formation enhances the affinity for HpHb and becomes even more critical for binding to isolated Hb. Conversely, Hb complexed with the Hp2-2 haplotype appeared to be bound and internalized equally well by both monomeric and oligomeric receptor forms.

Interestingly, both Zhuo et al.^1^ and Huang et al.^13^ converged on the idea that oligomerization may also mediate an autoinhibited receptor state. In the ligand-free form, CD163 can adopt a closed quaternary conformation in which the three rod-like SRCR1–4 segments converge to seal the ligand-binding pocket—a mechanism possible only in higher-order oligomers such as dimers or, more prominently, trimers. This self-shielded arrangement likely serves to prevent nonspecific ligand interactions while preserving receptor specificity.

Building on the findings discussed above, a number of key issues and avenues for future investigation arise regarding CD163 function and regulation.

CD163 has been previously characterized for its ability to interact with multiple ligands^15^, and the flexibility observed in the structural studies discussed here supports its capacity to accommodate diverse binding partners. Although the authors propose a straightforward internalization mechanism (i.e., ligand-induced endocytosis followed by lysosomal release, where pH and decrease of calcium concentration favor receptor monomerization and ligand dissociation) it remains unclear whether this model applies to all CD163 ligands. Small ligands such as TWEAK or TNF may follow this route, but the scenario is less evident and even less explored for larger ones, such as bacterial cells, for which CD163 acts as an innate immune sensor rather than an endocytic receptor^11^. The involvement of calcium in these interactions deserves further consideration, as several studies indicate that calcium may be essential for ligand binding^11,16^.

Another unresolved aspect concerns whether ligand binding triggers intracellular signaling, as previously suggested^17,18^, or simply facilitates cargo uptake. In vivo, CD163–HpHb interaction leads to HO-1 induction and IL-10 release, yet this anti-inflammatory response has been suggested to arise from heme degradation and CO production rather than canonical signal transduction^8^. It will be important to determine whether other ligands elicit distinct cellular responses and whether the trimeric, auto-inhibited form of CD163 could contribute to signal modulation.

In recent times, the role of sCD163 has emerged as important as that of the membrane-associated counterpart: it can bind to TWEAK and TNF, it modulates monocyte activation, and it enhances bacterial clearance^15^. This dual function underscores the complexity of CD163-mediated regulation at the interface of inflammation and immunity.

Given that calcium appears to stabilize SRCR-domain interactions, the structural insights gained from CD163 could extend far beyond this single receptor, offering a framework to understand how members of the SR family achieve ligand selectivity and signal regulation. The observed trimerization and calcium dependence may represent general mechanisms through which these receptors fine-tune their binding affinity and functional state, switching between inactive and active conformations in response to extracellular cues.

In recent years, CD163 has gained increasing attention as a prominent focus of research, with a surge of studies on its role in, e.g., secondary inflammation following intracerebral hemorrhage^19^, immunomodulation in the tumor microenvironment, and as a circulating biomarker of macrophage activation (sCD163)^20,21^. This growing attention reflects the receptor’s dual nature: protective in its role of clearing pro-oxidant Hb complexes, yet potentially detrimental when co-opted in malignant or chronic inflammatory settings. Future structural and cell-based investigations − particularly cryo-electron tomography on CD163-overexpressing cellular models − could provide a more direct insight of ligand recognition, receptor clustering, and internalization events in their native membrane context.

The future challenge that can be envisaged will be to harness the structural insights offered by these studies, i.e., receptor oligomerization and autoinhibition, to develop tailored therapies that selectively target specific functions while preserving others. The insights gained from CD163 may prove valuable for understanding how ligand heterogeneity has shaped the functional landscape of the SR superfamily, helping to distinguish their unique features from their shared functional principles.
